# The role of histone methyltransferases in therapeutic resistance of NSCLC

**DOI:** 10.1080/15592294.2025.2536786

**Published:** 2025-07-23

**Authors:** Fuze Zhu, Xudong Yang, Yanlong Yang, Xinghe Tong, Jie Jia, Xingkun Gu, Yunping Zhao, Xiaobo Chen

**Affiliations:** Department of Thoracic Surgery, First Affiliated Hospital of Kunming Medical University, Kunming, Yunnan, China

**Keywords:** Histone methylation, therapeutic resistance, non-small cell lung cancer, histone methyltransferases, EZH2

## Abstract

Conventional treatments, including chemotherapy, immunotherapy, targeted therapy and radiotherapy, are effective clinical strategies for non-small cell lung cancer (NSCLC) patients, which can significantly improve life quality and prolong survival time. However, the application of drugs in NSCLC patients inevitably leads to therapeutic resistance. In recent years, many studies have shown that histone methyltransferases (HMTs), including both protein arginine methyltransferases (PRMTs) and lysine methyltransferases (KMTs), play pivotal roles in tumor initiation, progression, and treatment resistance. This review synthesizes current insights into histone methylation dynamics driving therapeutic resistance, with a focus on key HMTs and their mechanisms. Additionally, we discuss the molecular mechanisms underlying histone methylation-mediated therapeutic resistance and potential therapeutic strategies targeting histone methylation for overcoming therapeutic resistance in NSCLC.

## Introduction

Lung cancer exhibits the highest global morbidity (12.4%) and mortality (18.7%) rates among all cancer types, with a generally poor prognosis, as evidenced by a 5-y survival rate of less than 20% in most countries [1,2]. Non-small cell lung cancer (NSCLC) is the predominant histological type, comprising approximately 85% of all lung cancer cases. NSCLC can be further categorized into histological subtypes, including adenocarcinoma (40%), squamous cell carcinoma (25%), and others [3]. Despite conservative treatment methods, such as chemotherapy, immunotherapy，targeted therapy and radiotherapy effectively prolong survival time of patients with advanced NSCLC, most of the patients inevitably acquire resistance leading to therapeutic failure [4]. Most of the molecular mechanisms of therapeutic resistance are still unknown, and only a portion of mechanisms have been extensively studied. For example, the T790M mutation is the main mechanism of resistance in first (gefitinib) and second (afatinib) generation epidermal growth factor receptor tyrosine kinase inhibitors (EGFR-TKIs) [5]. Recent research has increasingly demonstrated that epigenetic modifications play a significant role in the mechanisms underlying therapeutic resistance [6].

Epigenetic modifications – such as DNA methylation, histone modifications, and ncRNA regulation – alter gene expression by modulating transcription and translation without altering the DNA sequence [7]. As an important element of histone modifications, histone methylation can induce therapeutic resistance by activating bypass pathways (such as MET amplification) and downstream signaling pathways (such as PI3K/AKT axis) [8,9]. Histone methyltransferases (HMTs) classify two categories: protein arginine methyltransferases (PRMTs) and lysine methyltransferases (KMTs), which can catalyze histone methylations [10,11]. In this review, we systematically summary the mechanisms of HMTs leading to therapeutic resistance for NSCLC and reveal the potential of epigenetic therapies.

## HMTs

**Figure 1.**
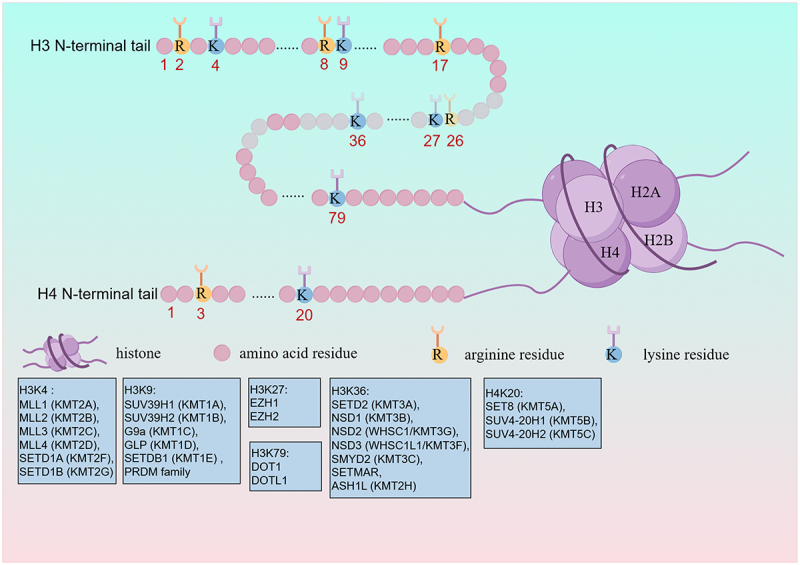
Histone Post-Translational Modifications (PTM) and associated enzymes: schematic representation of key K and R residues on the N-terminal tails of histones H3 and H4, along with their corresponding modifying enzymes.

Pairs of histone proteins H2A, H2B, H3, and H4 form histone octamers, which can be wrapped by approximately 147 base pairs (bp) DNA to composed Nucleosomes. The N-terminal tails of histone octamers are catalyzed by HMTs [12,13]. Among them, H3 and H4 histone tails on both lysine (K) residues and arginine (R) residues are the most common sites of histone methylation modification [14]. KMTs are divided into six types of methyltransferases based on their sites of methylation, including histone H3 lysine 4 methylation (H3K4), histone H3 lysine 9 methylation (H3K9), histone H3 lysine 27 methylation (H3K27), histone H3 lysine 36 methylation (H3K36), histone H3 lysine 79 methylation (H3K79) and histone H4 lysine 20 methylation (H4K20) [15]. These KMTs catalyze three methylation levels: mono-methylation (me1), di-methylation (me2), tri-methylation (me3) and each of them is linked to distinct functions [16]. As mentioned in previous reviews published, H3K4 methyltransferases include MLL1 (KMT2A), MLL2 (KMT2B), MLL3 (KMT2C), MLL4 (KMT2D), SETD1A (KMT2F), and SETD1B (KMT2G); H3K9 methyltransferases include SUV39H1 (KMT1A), SUV39H2 (KMT1B), G9a (KMT1C), GLP (KMT1D), SETDB1 (KMT1E) and PRDM family; H3K27 methyltransferases include EZH1 and EZH2; H3K36 methyltransferases include SETD2 (KMT3A), NSD1 (KMT3B), NSD2 (WHSC1/KMT3G), NSD3 (WHSC1L1/KMT3F), SMYD2 (KMT3C), SETMAR, and ASH1L (KMT2H); H3K79 methyltransferases include DOT1 (disruptor of telomeric silencing 1) or DOT1L (DOT1-like/KMT4); H4K20 methyltransferases induce SET8 (KMT5A), SUV4-20H1 (KMT5B), SUV4-20H2 (KMT5C) (Figure 1) [17,18]. The mechanism of these enzymes induced therapies for drug resistance is still unclear, and only a small portion has been studied, such as EZH1, EZH2, G9a, SET7/9, SMYD2, KMT5C (Tables 1 and 2). PRMTs are classified into three types: type I catalyzes arginine residues to asymmetric di-methylation (ADMA, me2a) and mono-methylation (MMA, me1), including PRMT1, PRMT2, PRMT3, PRMT4, PRMT6, and PRMT8; typeIIcatalyzes arginine residues to symmetric di-methylation (SDMA, me2s) and mono-methylation (MMA, me1), including PRMT5 and PRMT9; type III only catalyzes mono-methylation of arginine residues (MMA, me1), including PRMT7 [19]. In addition, most characterized histone arginine methylation occurs on H3R2, H3R8, H3R17, H3R26 and H4R3 (Figure 1) [20]. Of the nine PRMTs, two of them, PRMT1 and PRMT5, have been investigated in related to their potential roles in therapeutic resistance (Tables 1 and 2).

**Table 1.** The roles of histone methyltransferases and regulatory genes and pathways related in chemoresistance.

Methyltransferases	Roles in resistance	Drugs	Related genes and signal pathways	References
EZH2	Repressor	Cisplatin	LncRNA ACTA2-AS1, TSC2	[26]
EZH2	Promotor	Cisplatin	HOXB13	[24]
EZH2		Cisplatin	Linc00665, CDKN1C	[25]
EZH2		Mitoxantrone	MiR-200c，EZH2/E-cad	[27]
EZH2		Docetaxel	MiR-26a	[28]
EZH2/G9a		Cisplatin, Paclitaxel	SMAD4, RAF/ERK/c-Myc	[29]
EZH2		Cisplatin	LncRNA AFAP1‑AS1, PI3K/AKT	[30]
EZH2		Cisplatin	DDK1，Wnt	[31]
EZH2		Mitoxantrone	JAK/STAT	[32]
EZH2		Cisplatin	-	[33]
Set7/9		Doxorubicin	-	[35]
SMYD2		Cisplatin	P53	[37]
SMYD2		Cisplatin	Wnt/b-catenin	[38]
SMYD2		Doxorubicin	JAK-STAT	[40]
PRMT1		Cisplatin, Paclitaxel	FEN1	[41]
PRMT5		Etoposide, Cisplatin	-	[42]

**Table 2.** The roles of histone methyltransferases and regulatory genes and pathways related in EGFR-TKIs resistance.

Methyltransferases	Roles in resistance	Drugs	Related genes and signal pathways	References
EZH2	Repressor	Gefitinib	EZH2/TSC2/p-mTOR	[50]
EZH2		Gefitinib	MEOX2 and GLI1，EGFR/AKT/ERK	[52]
EZH2	Promotor	Gefitinib	LINC00665, PI3K/AKT	[8]
EZH2		Gefitinib	LINC00969, NLRP3/caspase-1/GSDMD	[46]
EZH2		Gefitinib	HOTAIR, P16, P21	[47]
EZH2		Gefitinib	lncRNA UCA1, CDKN1A	[49]
EZH2		Gefitinib	CASC9，DUSP1，EGFR/AKT/ERK	[53]
EZH2		Gefitinib	DUXAP10，OAS2	[55]
EZH2		Gefitinib	ERK1/2-EZH2/Snail/EGFR	[54]
EZH2/G9a		Erlotinib	SMAD4, RAF/ERK/c-Myc	[29]
EZH1		Erlotinib	miR-17-5p.	[57]
KMT5C		Erlotinib	LINC01510, MET	[59]
G9a		Erlotinib, afatinib, and osimertinib	STAT3, miR-145-5p, HER3	[60]
PRMT-1		Erlotinib	EMT	[58]

## HMTs and chemoresistance in NSCLC

**Figure 2.**
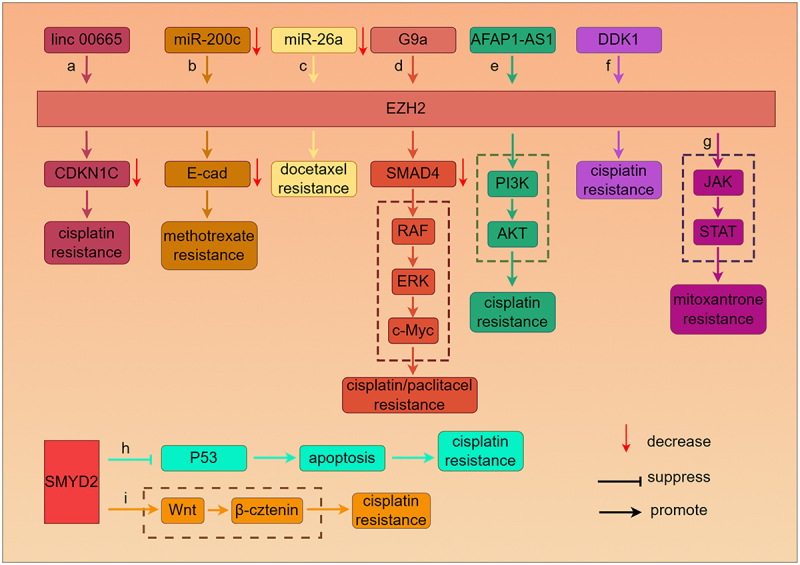
Molecular and signaling network underlying HMTs mediates chemoresistance in NSCLC. EZH2 mediates chemoresistance through interacting with ncRNA (a, b, c and e). EZH2 cooperates with G9a represses the expression of SMAD4, which activates the RAF/ERK/c-Myc signaling pathway to mediate chemoresistance (d). DDK1 mediates cisplatin resistance by up-regulating EZH2 (f). EZH2 directly activate JAK/STAT leading to mitoxantrone resistance (g). SMYD2 inhibits p53 expression and cell apoptosis, which mediates cisplatin resistance (h). SMYD2 activated Wnt/β-catenin signaling pathway leading to resistance to the cisplatin.

In the absence of a driver mutation, chemotherapy is one of the main treatment options for patients with advanced NSCLC who cannot undergo surgical resection [21]. However, chemoresistance is a critical challenge in the treatment of NSCLC [22]. EZH2, a component of the polycomb repressive complex 2 (PRC2), is a histone methyltransferase that catalyzes histone H3 lysine 27 tri-methylation (H3K27me3) [23]. HOXB13 is a transcription that can transcriptionally upregulate EZH2 by bonding to the EZH2 promoter, and then conferred resistance to cisplatin [24]. Many studies have shown that EZH2 mediates chemoresistance through interacting with ncRNA, including lncRNA and miRNA. Long non-coding RNA linc00665 recruits EZH2 to bind the promoter region of cyclin-dependent kinase inhibitor 1C (CDKN1C), which inhibits the transcription of CDKN1C and influenced the sensitivity of NSCLC cells to cisplatin (Figure 2(a)) [25]. LncRNA ACTA2-AS1 positively facilitates the cell apoptosis of cisplatin-resistant NSCLC cell lines. Overexpression of ACTA2-AS1 can recruit EZH2, which epigenetically suppresses the expression of TSC2 and revises the cisplatin resistance of NSCLC cells [26]. MiR-200c that function as a tumor suppressor gene regulates methotrexate sensitivity through inhibiting the expression of EZH2 and elevating the expression of E-cadherin. On the contrary, low level of miR-200c can mediate acquired resistance to methotrexate via the EZH2/E-cad pathway (Figure 2(b)) [27]. EZH2 is negatively regulated by miR-26a, which enhances the expression of EZH2 to mediate docetaxel resistance (Figure 2(c)) [28]. In addition, EZH2 can activate or suppress distinct signaling pathways leading to chemoresistance. EZH2 cooperates with G9a to induce drugs resistance through repressing the expression of tumor-suppressor gene SMAD4. The low expression of SMAD4 activates the RAF/ERK/c-Myc signaling pathway to mediate cisplatin, paclitaxel and erlotinib resistance (Figure 2(d)) [29]. The high level of lncRNA AFAP1‑AS1 contributes to the acquisition of malignant phenotypes of cisplatin resistance NSCLC Cells by interacting with EZH2 to activate PI3K/AKT pathway (Figure 2(e)) [30]. Dickkopf-1 (DDK1), a gene involved in Wnt signaling pathway, is coordinately up-regulated with EZH2 to drive a cisplatin refractory phenotype in NSCLC cell lines (Figure 2(f)) [31]. Cancer stem cells (CSCs) is involved in differentiation, proliferation and therapy resistance of cancer cells. EZH2 epigenetically activates the JAK/STAT signaling pathway, promoting cancer cell stemness and mitoxantrone resistance. (Figure 2(g)) [32]. Clinical studies have shown that overexpression of EZH2 often leads to platinum-based chemotherapy resistance and poorer prognosis in NSCLC patients [33].

SET7/9 is a histone methyltransferase that facilitates the mono-methylation of histone H3 at lysine 4 (H3K4me1) [34]. Alexandra Daks et al. detected that Set7/9 knock-out or effect of Set7/9-specific inhibitor could enhance the sensitivity of doxorubicin in NSCLC cells. Accordingly, it could be confirmed that doxorubicin resistance of NSCLC cells was Set7/9-dependent [35]. SMYD2 is also a histone methyltransferase that includes a SET domain and a MYND domain. The SET domain has lysine-specific methyltransferase activity, which can catalyze methylation of H3K36. While the MYND domain contains a zinc-finger motif able to catalyze non-histone proteins such as p53, PTEN [36]. Shang et al. revealed that SMYD2 targeted Lys-370 of p53 and transcriptionally down-regulating activity of p53, which inhibited cell apoptosis and mediated cisplatin-resistant malignant phenotypes in NSCLC cells (Figure 2(h)) [37]. Galván-Femenía et al. demonstrated that SMYD2 facilitated methylation of β-catenin for nuclear translocation and activated Wnt/β-catenin signaling pathway leading to a higher resistance to the cisplatin (Figure 2(i)) [38,39]. In addition, SMYD2 inhibitor BAY-598 can significantly enhance the sensitivity of the doxorubicin against NSCLC, through coordinately inhibited the JAK-STAT signaling pathway [40]. For the results of current studies, PRMTs can serve as promotors or repressors in chemoresistance. In 2020, He et al. studied that Flap endonuclease 1 (FEN1) played an important role in the DNA repair ability and apoptosis of lung cancer cells. PRMT1 was one of the arginine methyltransferases, which epigenetically up-regulated FEN1 leading to cisplatin and paclitaxel resistance by elevating the DNA repair ability and inhibiting the apoptosis of lung cancer cells [41]. In 2023, Shen et al. confirmed that PRMT5 was able to stimulate chemotherapy-induced neuroendocrine differentiation (NED) in NSCLC, which was an etoposide and cisplatin-resistant malignant phenotype [42].

The roles of HMTs and associated regulatory genes/pathways in NSCLC chemoresistance are summarized in Table 1.

## HMTs and EGFR-TKIs resistance in NSCLC

Epidermal growth factor receptor (EGFR) gene mutations are prevalent driver mutations in NSCLC, particularly adenocarcinomas [43]. A study shown that the EGFR mutation frequency has exceeded 50% in Asian patients with lung adenocarcinoma [44].

**Figure 3.**
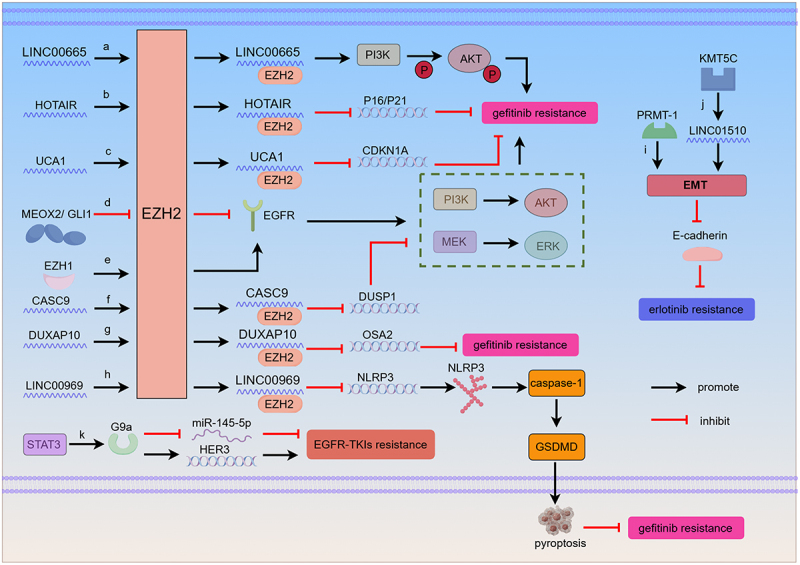
Molecular mechanisms of EGFR-TKIs resistance mediated by HMTs in NSCLC. LncRNAs and EZH2 epigenetic regulators: LINC00665, HOTAIR, UCAT, DUXAP10, and LINC00969 interact with EZH2 contributing to EGFR-TKIs resistance (a, b, c, g and h). MEOX2/GLI1 and EZH1 interacts with EZH2 mediating gefitinib resistance through activating EGFR and downstream PI3K/AKT/ERK pathways (d and e). EZH2 interacted with CASC9 mediates EGFR-TKIs resistance through inhibiting the expression of DUSP1 and activating downstream PI3K/AKT/ERK pathways (f). PRMT-1 and KMT5C mediate erlotinib resistance by activating EMT bypass pathway and repressing the expression of E-cadherin (i and j). STAT3-G9a axis induces EGFR-TKIs resistance through suppressing miR-145-5p and increasing HER3 expression (k).

Despite EGFR-TKIs significantly prolong survival time of patients with LUAD in primary, most of the patients inevitably acquire resistance during 9–13 months of treatment [8,45]. As reviewed in previous publications, EZH2 can induce gefitinib resistance by interacting with LINC00665, then activate downstream signaling pathways PI3K/AKT (Figure 3(a)) [8]. In 2023, Dai et al. found that NLRP3 played an important role in cell pyroptosis, which could inhibit the NLRP3/caspase-1/GSDMD-associated classical pyroptosis signaling pathways and reduce pyroptosis in tumor cells. LINC00969 collaborated with EZH2 to transcriptionally modulate the levels of H3K27me3 in the NLRP3 promoter region, thereby epigenetically suppressing NLRP3 expression, conferring an anti-pyroptotic phenotype, and enhancing TKI resistance in lung cancer (Figure 3(h)) [46]. P16 and P21 are two tumor suppressor genes that can regulate cell cycle progression. The interaction between HOTAIR, a long non-coding RNA Hox transcript antisense intergenic RNA, and EZH2 induces H3K27me3 in the promoter regions of p16 and p21, thereby inhibiting their expression and leading to cell cycle dysregulation and gefitinib resistance (Figure 3(b)) [47]. The cyclin-dependent kinase inhibitor

p21/WAF1/CIP1/CDKN1A is encoded by the CDKN1A gene, which can inhibit cell cycle progression and lead to cell cycle arrest by suppressing cyclin-dependent kinase (CDK) [48]. Xu et al. demonstrated that EZH2 binds to lncRNA UCA1, inducing gefitinib resistance via epigenetic silencing of CDKN1A. (Figure 3(c)) [49]. In 2020, Cao et al. confirmed that the downregulation of EZH2 could epigenetically regulate autophagy via EZH2/TSC2/p-mTOR signaling pathway to mediate EGFR-TKIs resistance [50]. Both PI3K/AKT and MEK/ERK signaling pathways are two EGFR-TKIs resistance-related signaling pathways [51]. Peralta-Arrieta et al. hypothesized that transaction factors both MEOX2 and GLI1 could decrease epigenetic marks EZH2/H3K27me3 at EGFR-gene promoter, which leaded to EGFR overexpression to activate EGFR/AKT/ERK signaling pathway inducing EGFR-TKIs resistance (Figure 3(d)) [52]. Furthermore, Chen et al. assumed that EZH2 interacted with CASC9 to occupy the promoter regions of tumor suppressor DUSP1, which epigenetically inhibited the expression of DUSP1 mediating EGFR-TKIs resistance through increasing EGFR, ERK and AKT signaling pathways (Figure 3(f)) [53]. In 2024, Tang et al. confirmed that the ERK1/2-EZH2/Snail/EGFR signaling pathway plays a vital role in drugs resistance. ERK1/2 induces the overexpression of EZH2, which can mediate gefitinib resistance by epigenetically upregulating Snail and EGFR (Figure 3(e)) [54]. As a tumor suppressor, OAS2 is related to gefitinib resistance that DUXAP10 recruits EZH2 epigenetically silencing OAS2 (Figure 3(g)) [55].

EZH1 is another enzymatic component of PRC2, which catalyzes histone H3K27 tri-methylation (H3K27me3) to inhibit the expression of PRC2 target genes [56]. Overexpression of EZH1 mediates erlotinib resistance via down regulations of upstream miR-17-5p [57]. Epithelial-mesenchymal transition (EMT) is a vital bypass pathway leading to EGFR-TKIs resistance. PRMT-1, a EMT regulator, can mediate erlotinib resistance by activating EMT bypass pathway and repressing the expression of E-cadherin (Figure 3(i)) [58]. In addition, silence of KMT5C is related to erlotinib resistance by promoting overexpression of the oncogene LINC01510. LINC01510 is a regulator of mesenchymal-epithelial transition (MET) and positively regulates elevation of MET to mediate erlotinib resistance (Figure 3(j)) [59]. Chang et al. detected that the overexpression of G9a, positively regulated by STAT3, could induce EGFR-TKIs (gefitinib, afatinib, and osimertinib) resistance through transcriptionally suppressing miR-145-5p but increasing HER3 expression (Figure 3(k)) [60].

The roles of HMTs and regulatory genes and pathways involved in EGFR-TKIs resistance in NSCLC are recorded in Table 2.

## HMTs and immune evasion in NSCLC

For unselected NSCLC patients immunotherapy with immune checkpoint inhibitors (ICIs), including Anti-Programmed Cell Death Protein 1 (anti-PD-1) and Anti-Programmed Death-Ligand 1 (anti-PD-L1) is a promising treatment strategy [61]. Nevertheless, immune evasion is a challenge for immunotherapy [62]. Studies have shown that HMTs mediate immune escape through various mechanisms. A predominant strategy involves modulating immune cell functionality and signaling pathways, ultimately leading to TME dysregulation. For example, EZH2 might facilitate tumor immune evasion by T-cell exclusion and dysfunction. T-cell exclusion can mediate tumor immune evasion by decreasing the infiltration of immune cells and promoting the infiltration of immunosuppressive cells, including myeloid-derived suppressor cells (MDSC), cancer-associated fibroblasts (CAFs), and regulatory T cells (Tregs). Moreover, EZH2 is positively related to the infiltration of Tregs, MDSC, and CAFs in multiple cancers. Furthermore, in most tumors, EZH2 expression is positively correlated with T-cell dysfunction, which facilitates the immune evasion of tumor cells leading to drug resistance [63]. In addition, Guo et al. detected that EPIC1 could epigenetically silence IFNGR1 and antigen presentation genes via binding with EZH2. Subsequently, the reduction of IFNGR1 inhibited the IFN-γ–JAK – STAT1 signaling and antigen presentation, which resulted in tumor cells evading the immune surveillance and developing resistance to immune checkpoint blockade therapy [64]. In 2019, Zhao et al. EZH2 promotes the establishment of an immunosuppressive microenvironment through epigenetically upregulating the hypoxia-inducible factor alpha (HIF-1α) to stimulate PD-L1 expression [65].

**Figure 4.**
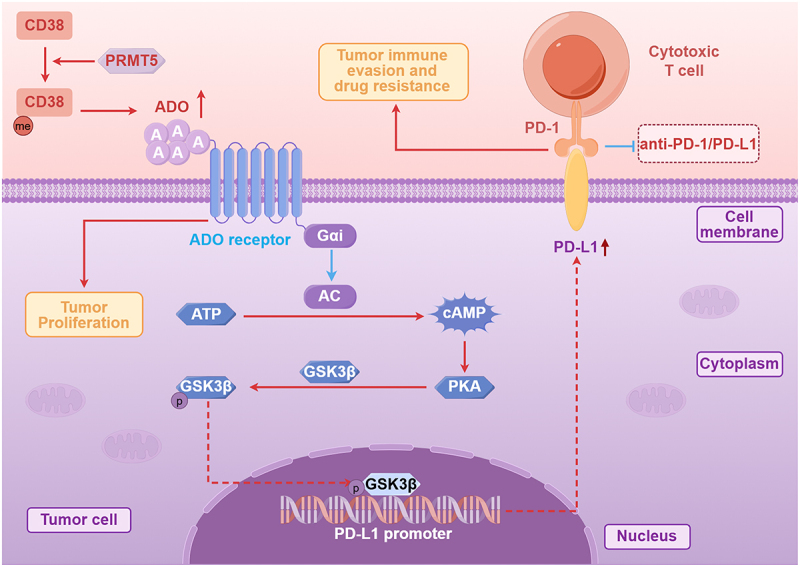
Schematic illustration of tumor immune evasion mechanisms involving the CD38/PRMT5/ADO pathway. PRMT5 drives tumor proliferation and immune evasion through epigenetic regulation and synergizes with CD38-mediated ADO- cAMP-PKA axis, alongside blockading programmed cell Death protein 1 or programmed Death-ligand 1 (PD-1/PD-L1) checkpoint and suppressing T cell activity.

PRMT family members facilitate the establishment and maintenance of drug tolerance in cancer cells through diverse mechanisms, such as dysregulating drug efflux transporters, augmenting DNA repair pathways, inducing autophagy, sustaining CSCs properties, driving EMT, and perturbing the tumor microenvironment (TME) [66]. CGAS-STING Pathway play an important role in tumor immune. The stimulator of the interferon gene (STING), a transmembrane protein residing in the endoplasmic reticulum, is activated by cytosolic double-stranded DNA (dsDNA). Cyclic GMP-AMP synthase (cGAS) functions as a critical sensor for dsDNA and is essential for STING activation. Activation of cGAS-STING pathway triggers the interferon regulatory factor 3 (IRF3)-TANK binding kinase 1 (TBK1) signaling cascade, promoting the production of cytokines and type I interfere [67]. As a suppressor of cGAS-STING pathway, PRMT5 attenuates type I interferon (IFN) and chemokine production through di-methylating cGAS complex components interferon gamma inducible protein 16 (IFI16) and suppresses the invasion of CD4+ T cells and CD8+ T cells into rumors to support immune evasion [68]. Huang and colleagues also demonstrated that PRMT1 facilitates tumor immune evasion by suppressing the cGAS/STING signaling pathway via methylation-mediated cGAS inactivation [69]. Furthermore, PRMT5 catalyzes symmetric dimethylation at the R58 residue of CD38, promoting extracellular adenosine (ADO) production. Subsequenly, ADO combines with adenosine receptors to enhance cyclic AMP (cAMP), and then cAMP stimulates protein kinase A (PKA), which makes glycogen synthase kinase 3 beta (GSK3β) phosphorylate and inhibits the function of GSK3β [70,71]. Ultimately, inhibition of GSK3β upregulates the expression of PD-L1 in tumor cells that facilitating tumor immune escape [72–74]. All in, PRMT5 interacts with CD38 activating the phosphorylation of GSK3β through the ADO-cAMP-PKA axis, augmenting stabilization of PD-L1 expression and subsequently inhibiting CD8+ T cell-mediated tumor cell killing, supporting tumor cells to evade immune surveillance (Figure 4) [75]. Hu et al. confirmed that targeting PRMT5 in cancer patients with inhibitors hindered lung cancer progression, but conversely augmented PD-L1 expression that inhibited the cytotoxicity of CD8+ T cells resulting in immune resistance [76]. PRMTs actively remodel the TME by mediating dynamic crosstalk between tumor cells and stromal elements, while concurrently facilitating immune evasion. This orchestration of the TME further fosters drug resistance [77].

The ablation of MLL4 elevates the double-stranded RNA (dsRNA)-interferon response and gasdermin D (GSDMD)-mediated pyroptosis through decommissioning enhancers to suppress the expression of RNA-induced silencing complex (RISC) and DNA methyltransferases. Thus, targeting MLL4 might be a potential way to overcome immunotherapeutic resistance in cancer patients [78]. Phosphorylated SET1 histone methyltransferase (SET1A) mediates the monomethylation of Yes-associated protein (Yap), which promotes Yap to accumulate in the nuclear and transcriptionally upregulates fibrinogen-like protein 1 (FGL1) expression. Subsequently, overexpression of FGL1 binds with lymphocyte activation gene 3 (LAG-3) receptors on the surface of T cells and hampers T-cell functions evading immune surveillance [79–81].

The roles of HMTs and regulatory genes and pathways involved in immune evasion in NSCLC are recorded in Table 3.

**Table 3.** The roles of histone methyltransferases and regulatory genes and pathways related in immune evasion.

Methyltransferases	Roles in resistance	Related genes and signal pathways	References
PRMT5	Repressor	PD-L1, CD8+ T	[75]
EZH2	Promotor	Tregs, MDSC, and CAFs	[63]
EZH2		EPIC1, IFNGR1， IFN-γ–JAK – STAT1	[64]
EZH2		HIF-1α, PD-L1	[65]
PRMT5		cGAS-STING, IRF3, TBK1, IFN, IFI16, CD4+ T, CD8+ T	[67,68]
PRMT5		CD38，GSK3β，PD-L1， CD8+ T，ADO-cAMP-PKA	[70,75]
MLL4		GSDMD，RISC	[78]
SET1A		Yap, FGL1, LAG-3	[79–81]

## HMTs and radioresistance in NSCLC

Radiotherapy is an important means of cancer treatment, but radiotherapy resistance has been considered as one of the main reasons for clinical treatment failure [82]. HMTs play an important role in cancer radiotherapy resistance by catalyzing histone methylation, regulating gene expression, DNA damage repair, cell cycle regulation, and tumor stem cell characteristics [83]. Cheng et al. demonstrated that PRMT1 catalyzed methylation of plakophilin 2 (PKP2) that recruited USP7 and stabilize β-catenin to enhance the transcription level of LIG4. Concomitantly, overexpression of LIG4 induced radioresistance through nonhomologous end-joining (NHEJ) repairing in lung cancer. In brief, PRMT1-mediated radioresistance depended on PKP2/β-catenin/LIG4 axis [84]. Aberrant expression of G9a induces radioresistance in lung cancer patients by decreasing the expression of tumor suppressor gene CCDC8 [85]. Posttranslational arginine methylation of M×i1by PRMT5 facilitates the ubiquitination and degradation of M×i1by binding of the β-Trcp ligase, causing radio resistance in lung cancer [86].

## Conclusion

The intricate role of HMTs in driving therapeutic resistance across diverse treatment modalities in NSCLC underscores their centrality as both culprits and therapeutic targets in oncology. This review synthesizes compelling evidence that HMTs, through dynamic epigenetic reprogramming, orchestrate resistance mechanisms that undermine chemotherapy, EGFR-TKIs, immunotherapy, and radiotherapy. Notably, enzymes such as EZH2 and PRMT5 emerge as master regulators, hijacking non-coding RNAs (e.g., LINC00665, ACTA2-AS1), rewiring oncogenic pathways (e.g., PI3K/AKT, Wnt/β-catenin), and sculpting immunosuppressive microenvironments to confer survival advantages to NSCLC cells. Beyond their canonical roles in histone modification, HMTs exhibit pleiotropic functions, such as methylating non-histone substrates (e.g., p53, β-catenin) and modulating DNA repair machinery, thereby expanding their influence over tumor plasticity and therapy evasion.

While this review focuses on the resistance mechanisms of EZH2 and PRMT5 in NSCLC, it is critical to acknowledge that HMTs constitute a large family with diverse members whose roles in therapeutic resistance remain underexplored. Notably, the functional complexity of HMTs extends beyond individual enzyme activities, as emerging evidence suggests potential synergistic or antagonistic interplay between different members (e.g., EZH2-PRMT5 crosstalk) in shaping resistance phenotypes. However, current studies predominantly investigate isolated HMTs, leaving their dynamic interaction networks. For instance, the contributions of KMT5C in EGFR-TKI resistance and SMYD2 in DNA damage response remain poorly characterized, highlighting significant knowledge gaps in our understanding of HMT networks. Future research should prioritize systematic mapping of HMT interactomes through multi-omics integration and functional genomics approaches to dissect their combinatorial roles in resistance evolution.

A novel perspective lies in the spatiotemporal heterogeneity of HMT activity. Emerging data suggest that resistance is not static but evolves dynamically, driven by context-dependent HMT interactions with tumor-intrinsic factors (e.g., stemness programs) and extrinsic pressures (e.g., immune surveillance). For instance, EZH2’s dual role in suppressing tumor suppressor genes (e.g., CDKN1A, NLRP3) while activating pro-survival signals (e.g., Snail/EGFR) highlights its adaptability as a molecular switch in resistance trajectories. Similarly, PRMT5’s crosstalk with adenosine signaling to stabilize PD-L1 unveils a previously underappreciated axis linking metabolism, epigenetics, and immune evasion.

Therapeutic innovation must pivot toward precision epigenetic targeting. While inhibitors of EZH2 (e.g., tazemetostat) and PRMT5 (e.g., GSK3326595) show promise, their efficacy is hindered by compensatory mechanisms and off-target effects. Future strategies could exploit synthetic lethality by combining HMT inhibitors with DNA damage agents (e.g., PARP inhibitors) or immunotherapies to dismantle redundant survival pathways. Additionally, leveraging single-cell multi-omics to map HMT-driven clonal evolution during treatment could identify resistance-specific epigenetic vulnerabilities, enabling timed interventions.

Finally, TME emerges as a critical battleground. HMTs like EZH2 and MLL4 regulate immune cell infiltration and function, suggesting that epigenetic therapies could ‘re-sensitize’ tumors to checkpoint inhibitors by reprogramming the TME. For example, disrupting PRMT5-mediated CD38 methylation may reverse adenosine-driven immunosuppression, while MLL4 inhibition could amplify immunogenic pyroptosis via dsRNA sensing. These approaches align with the growing paradigm of epigenetic immunomodulation, where HMT targeting reshapes both tumor and immune landscapes.

In conclusion, HMTs are not passive bystanders but architects of NSCLC resilience. Deciphering their context-dependent roles and developing mechanism-driven combinatorial regimens will be pivotal to overcoming therapeutic resistance. By integrating epigenetics into the precision oncology framework, we can transform NSCLC from a refractory malignancy into a tractable disease. Targeting HMTs could be a potential therapeutic strategies for overcoming therapeutic resistance in NSCLC.

## Data Availability

Data sharing is not applicable to this article as no new data were created or analysed in this study.
